# Context-dependent generalization

**DOI:** 10.3389/fnhum.2013.00171

**Published:** 2013-05-06

**Authors:** Jordan A. Taylor, Richard B. Ivry

**Affiliations:** ^1^Department of Psychology, Princeton UniversityPrinceton, NJ, USA; ^2^Department of Psychology, University of CaliforniaBerkeley, CA, USA; ^3^Helen Wills Neuroscience Institute, University of CaliforniaBerkeley, CA, USA

**Keywords:** motor control, motor learning, motor adaptation, models, theoretical, generalization (psychology)

## Abstract

The pattern of generalization following motor learning can provide a probe on the neural mechanisms underlying learning. For example, the breadth of generalization to untrained regions of space after visuomotor adaptation to targets in a restricted region of space has been attributed to the directional tuning properties of neurons in the motor system. Building on this idea, the effect of different types of perturbations on generalization (e.g., rotation vs. visual translation) have been attributed to the selection of differentially tuned populations. Overlooked in this discussion is consideration of how the context of the training environment may constrain generalization. Here, we explore the role of context by having participants learn a visuomotor rotation or a translational shift in two different contexts, one in which the array of targets were presented in a circular arrangement and the other in which they were presented in a rectilinear arrangement. The perturbation and environments were either consistent (e.g., rotation with circular arrangement) or inconsistent (e.g., rotation with rectilinear arrangement). The pattern of generalization across the workspace was much more dependent on the context of the environment than on the perturbation, with broad generalization for the rectilinear arrangement for both types of perturbations. Moreover, the generalization pattern for this context was evident, even when the perturbation was introduced in a gradual manner, precluding the use of an explicit strategy. We describe how current models of generalization might be modified to incorporate these results, building on the idea that context provides a strong bias for how the motor system infers the nature of the visuomotor perturbation and, in turn, how this information influences the pattern of generalization.

## Introduction

Generalization following practice of a new motor task has provided an important tool for evaluating the specificity of learning. By examining whether or not the effects of training extend to untrained movements and novel contexts, we gain insight into the representational changes that have occurred during learning (Poggio and Bizzi, 2004). Generalization designs have been widely used in studies of sensorimotor adaptation with the pattern of generalization providing clues as to how movement is computed and updated through learning (Ghahramani et al., 1996; Thoroughman and Shadmehr, 2000; Donchin et al., 2003; Thoroughman and Taylor, 2005). These studies have revealed that the motor system does not learn by a simple look-up table (Atkeson, 1989; Conditt et al., 1997; Mussa-Ivaldi, 1999), but rather builds an internal model to approximate the sensorimotor mapping required for controlling reaches in a particular environment.

One common method for studying adaptation is to perturb the visual feedback, either by imposing a lateral translation (e.g., prism glasses) or an angular deviation (e.g., visuomotor rotation). These perturbations introduce an error between the expected and actual visual feedback, a signal that is used to modify an internal model. In generalization studies of visuomotor adaptation, training is restricted to movements in a particular direction or some subregion of the workspace, followed by testing with movements in novel directions or regions of the workspace (Pine et al., 1996; Krakauer et al., 2000).

The form and extent of generalization show distinct characteristics for these two types of perturbations. Following a translation, generalization is broad, spanning the entire workspace (Ghahramani et al., 1996). In contrast, generalization following visuomotor rotation has been found to be relatively narrow, with strong generalization for movements similar to the training direction and falling off rapidly as the probe directions deviate from this direction. When considered in polar coordinates, the degree of generalization falls to approximately 25% for movements 45° away from the training location (Krakauer et al., 2000). Nonetheless, most studies have found a small degree of generalization throughout the entire workspace (Pine et al., 1996; Krakauer et al., 2000; Tanaka et al., 2009), although the magnitude at distant locations is generally less than what is observed with translational shifts (Ghahramani et al., 1996).

**Figure 1:**
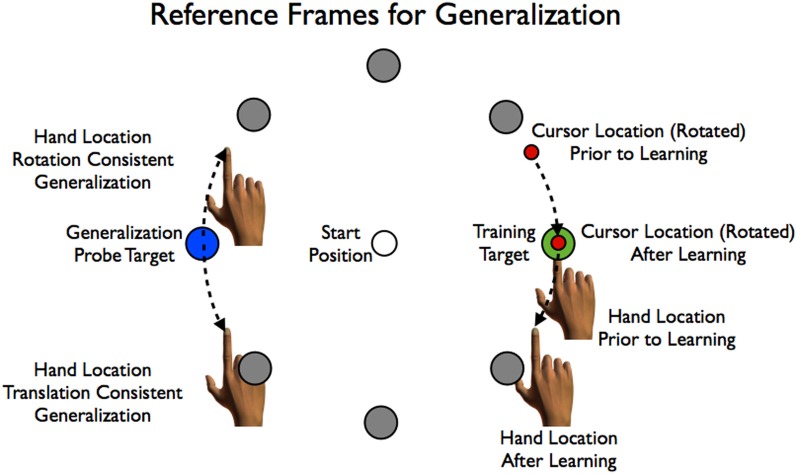
**Possible patterns of generalization.** A 30° rotation on the cursor (red circle) is imposed during movements to the training target at 0° (green). Generalization is tested at a probe target at 180° (blue). Generalization consistent with learning a rotation would appear as a clockwise shift in hand angle for movements to the probe target. Generalization consistent with learning a translation would appear as a counterclockwise shift in hand angle.

Studies in which participants adapt to a visuomotor rotation have, for the most part, reported generalization patterns that are consistent with the direction of the rotation. However, the results of two recent studies suggest that generalization may entail another component, one that indicates that participants may be inferring a translational shift, either in addition to a rotation (Brayanov et al., 2012), or in lieu of a rotation (Taylor et al., 2013). Taylor et al. (2013) trained participants to reach to a target to the right of the starting position, imposing a counterclockwise (CCW) rotation that was counteracted by movements in the clockwise, or downward direction. When the participant was provided with full, online visual feedback, reaches to novel targets positioned in the opposite side of the workspace showed trajectories that were also deviated in the clockwise (CW) direction (now upward), consistent with what would be expected if a rotational had been learned (Figure 1). In contrast, when only endpoint feedback was provided during training, the trajectories to the novel locations were deviated in the counterclockwise direction, or downward, consistent with a translational perturbation (Figure 1). Thus, training under different forms of feedback led to very different patterns of generalization. Importantly, modeling of these different patterns of generalization revealed an alternative account of the broad, albeit modest, generalization observed in the online feedback condition. This generalization could be due to incidental training for movements in the direction of the generalization targets that occurred as the participants either made corrective movements to the training target or moved back to the starting location during the training phase of the experiment.

The behavioral and theoretical work on generalization have focused on how an internal model is modified, based on the tuning properties of the motor system and the form of the error signal. Ignored in this discussion is how the environmental context may also influence learning and, as such, constrain generalization. This is surprising given that contextual effects have been shown to provide a powerful source of constraint in a wide range of motor tasks (Hommel, 1993; McNevin et al., 2000; Mechsner et al., 2001; Ivry et al., 2004). In sensorimotor adaptation studies, the context can be defined by the layout of the target locations. For example, the targets might be limited to a single location, constrained to fall within a limited part of space (e.g., fixed radial distance from a start location), or broadly distributed across the workspace. Interestingly, previous studies of generalization have always confounded the arrangement of the target locations and the type of visual perturbation in that experimenters have employed a reaching environment consistent with the perturbation. In studies where the perturbation was a rotation, the targets were arranged in a circular manner. In contrast, in studies where the perturbation involved a translation, the targets were arranged in a rectilinear manner (Ghahramani et al., 1996). Thus, there has always been a confound between the form of the visual errors (rotation or translation) and the arrangement of the targets (circular or rectilinear). This confound makes it impossible to evaluate the relative contribution and interaction of these factors with respect to generalization, and in particular, to understand why the extent of generalization varies for different perturbations (e.g., narrow for rotation, broad for translation).

The present study was designed to untangle this confound. We first conducted an experiment in which the context and perturbation were consistent, similar to what has been implicit in previous studies of generalization. One group of participants learned to overcome a rotation with targets that were arranged on a circle while a second group of participants learned to overcome a translational shift with targets that were arranged on a set of lines. Our goal here was to replicate previous work, but in a single experiment in which all other factors were identical for the two groups. In a second experiment, we swapped the context for the two types of perturbations, creating conditions in which these two factors were inconsistent with one another. One group of participants learned to overcome a rotation with targets that were arranged on a line while a second group of participants learned to overcome a translational shift with targets that were arranged on a circle. Comparing the results, both within and between these experiments, should allow us to assess the relative contribution of context and visual error signals to generalization.

## Materials and methods

### Participants

Forty participants (24 females/16 males, ages 18–24) were recruited from the Department of Psychology research participation pool at the University of California, Berkeley. Participants received class credit for participation. All participants were right handed, measured by the Edinburgh handedness inventory (Oldfield, 1971). Sixteen participants participated in experiment one, sixteen in experiment two, and eight in experiment three. The experimental protocol was approved by the institutional review board of the University of California, Berkeley.

### Experimental apparatus

Participants held onto a digitizing pen and made center-out reaching movements to visually displayed targets (7 mm diameter) by sliding the pen across a digitizing tablet (Intuous 3, Wacom, Vancouver, WA, USA). The targets and other task stimuli were displayed on a 15-in., 1280 × 1024 pixel resolution LCD monitor, mounted 25.4 cm above the tablet. The monitor was oriented horizontally to match the plane of the tablet. This configuration occluded vision of the hand and feedback of hand position was limited to a small cursor (3.5 mm diameter); when veridical, the feedback cursor was directly above the hand. The experimental task was implemented using custom software written in Python (open source) and run on a laptop computer.

### Experiment 1

**Figure 2:**
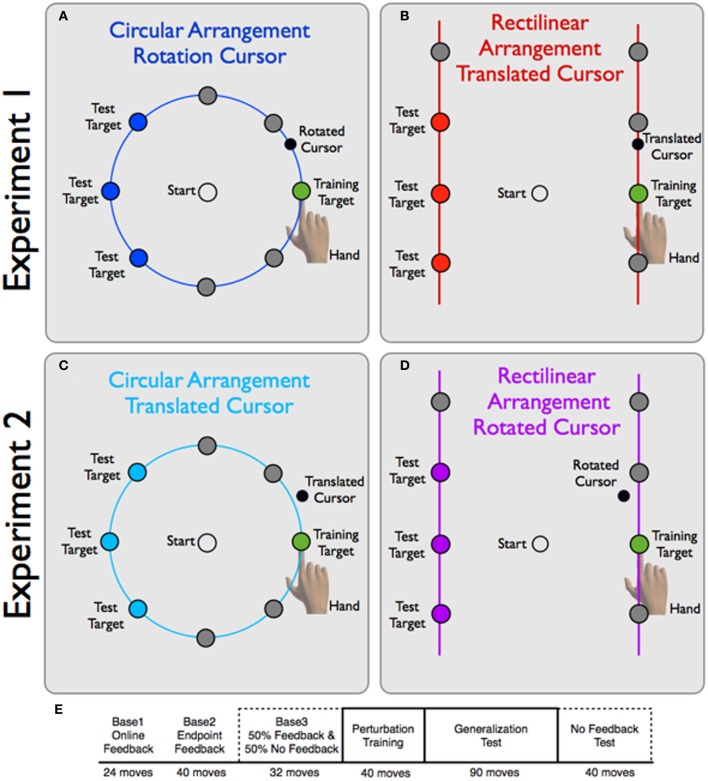
**Experimental design for Experiments 1 and 2. (A,B)** Experiment 1: Participants in the CircleRotation group **(A)** viewed a blue ring. A target could appear at one of eight locations. During the training block, reaches were limited to the training target location and the visual perturbation was a 30° CCW rotation. The LineTranslation group **(B)** viewed two vertically oriented red lines, with four target locations on each vertical line. The visual perturbation here was a 4 cm vertically-oriented visual shift. **(C,D)** Experiment 2: for the CircleTranslation group, targets were presented on a blue ring **(C)** and the visual perturbation for the training target was a vertical shift of 4 cm. For the LineRotation group, targets were presented on two vertical lines and the perturbation was a 30° CCW rotation. Note that the endpoint feedback for both groups generally fell off of the contextual boundary. **(E)** In Baseline blocks and the No Feedback blocks, all target locations were equally probable. During the Training block, only the training target location (0°, green target) was present. In the Test block, the training target location and the probe target locations (blue for circular arrangement and red for rectilinear arrangement) were equally probable. After the Baseline blocks, visual feedback was only provided on trials to the training target location.

The 16 participants were assigned to one of two experimental groups (Figure 2). For the CircleRotation group, a circular ring (7 cm radius) was always visible on the screen. The visual target could appear at one of eight locations on the ring, with polar angles of 0°, 45°, 90°, 135°, 180°, −135°, −90°, and −45°. For the LineTranslation group, two vertical lines were always visible, displaced 7 cm to the left and right of the starting position. A single target could appear at one of eight possible locations on the lines, four per line. The targets were equally spaced along the lines, and when defined in polar coordinates, were at 0°, 35.2°, 54.7°, 125.3°, 144.7°,180°, −144.7°, and −35.2°. Since the targets were not arranged in a circular manner, the distances to targets varied across the four target pairs (leftward or rightward amplitudes, from bottom to top of 8.57, 7.0, 8.57, and 12.12 cm). Note that the locations were chosen such that the second target location from the bottom on each side was co-linear with the starting position (Figure 2), and the neighboring targets were within 10° of corresponding target positions for the CircleRotation group.

On each trial, a single target was presented and the participant was instructed to make a fast reaching movement, “slicing” through the target. Although the movements terminated beyond the targets, endpoint feedback was limited in the main part of the experiment to the appearance of a red cursor that appeared along the contextual landmark (circular ring or vertical lines). The feedback cursor remained visible for 2 s. To motivate the participants to move quickly, a pleasant “ding” sound was played whenever the target amplitude was reached within 500 ms. If the movement time exceeded this criterion, an aversive “buzz” sound was presented. At the end of the feedback period, the feedback cursor was replaced by a ring with a diameter corresponding to the distance between the hand and starting position. By moving toward the starting circle, this ring became progressively smaller. When the hand was within 1 cm of the starting circle, the ring was transformed into the white feedback cursor, allowing the participant to position the hand within the starting circle. This form of feedback provided a way to guide the participant back to the starting position without providing information about the visuomotor perturbation (e.g., rotation or translation, see below). The participant was required to keep the cursor within the starting position for 1 s, at which time the next target appeared. Feedback of the cursor was removed when the position of the hand exceeded 1 cm from the starting position. While we emphasized movement speed, we did not put any constraint on reaction time. Movements were generally initiated within 500 ms.

The experimental session consisted of 266 reaches, divided into six blocks (Figure 2—bottom row). The first 24 trials (Base1) were designed to familiarize participants with the experimental task and the guidance method for returning to the starting position. Veridical online feedback was presented during the outbound portion of the movement until the hand passed through the ring or line, for the circular and rectilinear contexts, respectively. Each target was presented three times. For the next 40 trials (Base2), the online feedback was replaced by endpoint feedback. This was followed by a final baseline block of 32 trials (Base3) during which the endpoint feedback was only presented on 50% of the trials. A pseudorandom procedure was employed such that, for each target location, endpoint feedback was presented on two trials and withheld on two trials. Participants were informed that feedback would be withheld on half of the trials. On these trials, the auditory feedback concerning movement time also served as a cue that the movement had reached the required target amplitude. We included these trials because we wanted to familiarize the participants with the no-feedback procedure that would be critical for the assessment of generalization.

Participants then completed a 40-trial visuomotor perturbation training block (Training). In this block, all reaches were to the 0° location (target located directly to the right of the starting position) and the visual feedback was perturbed. For the CircleRotation group, the perturbation consisted of a 30° counterclockwise (CCW) rotation of the feedback cursor relative to true hand position. For the LineTranslation group, the perturbation consisted of an upward 3.5 cm vertical shift relative to true hand position. The magnitude of this vertical shift was chosen to equal the angular distortion induced by a 30° rotation for a 7 cm movement to the 0° target location. Endpoint feedback was provided on all trials. We chose to use endpoint feedback because a translational perturbation involving a constant shift relative to hand position would introduce a discontinuity with online feedback (e.g., the cursor would jump the distance of the perturbation at movement onset). Participants were not informed of the perturbation, nor that the target would always appear at the same location.

Generalization was tested in the last two blocks. In the Test block, reaches to the training location (0°) were interleaved with reaches to three of the target locations (those corresponding to 135°, 180°, and −135° in the CircleRotation group and the targets approximating these positions in the LineTranslation group, see Figure 2). These three probe locations were chosen because they would be the most informative for determining the extent and form of generalization. Endpoint feedback was only provided for reaches to the training location; all reaches to the three probe locations were performed without any visual feedback. The Test block consisted of 90 movements, 45 to the training location and 15 to each of the three probe locations. Thus, feedback was provided on 50% of the trials. The trial sequence was pseudorandomly distributed such that, for every four movements, two were to the training location and two were to probe locations.

The second generalization block (No Feedback) consisted of 40 trials, five to each of the eight target locations. No visual feedback was presented on any of these trials, including those in which the target appeared at the training location. This block provided a full assessment of the generalization function.

Participants completed the series of six blocks in approximately 45 min.

### Experiment 2

To unconfound context and perturbation, we repeated the procedure of Experiment 1, but now employed an inconsistent mapping (Figure 2) by assigning the two perturbations to the opposite context. Thus, a rotation was employed in the rectilinear context, while a translation was employed in the circular context. Sixteen naive participants were assigned to one of two experimental groups. For the CircleTranslation group, targets appeared on a circular ring, but the perturbation, when present, was a 3.5-cm upward, vertical translation. In this condition, the feedback cursor at the onset of the training block was usually displaced outside of the circular ring. In contrast, in the LineRotation group, targets appeared on the vertical lines, but the visual perturbation was a 30° CCW rotation. Here, the feedback cursor at the onset of the training block was usually displaced inside of the vertical line. The organization of the 266 trials was identical to that of Experiment 1.

### Experiment 3

Various lines of evidence indicate that sensorimotor learning entails the operation of multiple learning processes. These processes can vary in terms of the weight they give to the error signal, how they decay over time, and their accessibility to awareness. We focus on the awareness issue in a third experiment, given that the broad generalization observed with a visuomotor translation might be taken to reflect the operation of a process not specific to adaptation *per se*, but one that might result from the generic application of a strategy. We imposed the visuomotor perturbation in a gradual manner since this method has been shown to constrain learning to processes associated with adaptation of a visuomotor mapping (Kagerer et al., 1997; Saijo and Gomi, 2010; Taylor et al., 2011). We limited testing to the translation condition to ask if the broad generalization observed with this kind of perturbation was eliminated when strategic processes were excluded.

Eight naive participants were trained with the rectilinear context. The translational shift was introduced in small increments, increased linearly from 0 cm to 3.5 cm over the course a 160 trial training block (a shift of 0.023 cm or 0.188° per trial for the first 152 trials, then held constant over the last 8 trials). The structure of the baseline blocks and generalization blocks was the same as in Experiments 1 and 2.

### Data analysis

Kinematic and statistical analyses were performed with Matlab (MathWorks, Natick, MA). To assess adaptation and generalization, we focused on the angular difference between the target location and the hand position when the hand intersected the circular ring or the vertical line. Each movement trajectory, regardless of the actual target location, was rotated to a common axis such that the target location was at 0°. A straight line was connected between the starting position and the actual hand position, and we computed the angle between this line and the 0° reference line. With this convention, positive angles indicate a positive deviation (CCW) along the *y*-axis and negative angles indicate a negative deviation (CW) along this axis.

To assess performance prior to the introduction of the perturbation, we performed two separate analyses on reaches made during the Base3 block. First, the endpoint hand angles, averaged over all target locations, were calculated for each participant. For Experiments 1 and 2, these values were submitted to a two-sample *t*-test to determine if there were significant differences between groups. In addition, the movement time and reaction time data were analyzed to see if these variables were influenced by the two contexts.

Second, we performed a more restricted analysis on the reaches in Base3 to the three probe locations since these will be of greatest interest in our assay of generalization. The average endpoint hand angle was calculated separately at each probe location. These values were submitted to a mixed-model repeated measures ANOVA with the within-participant factor, Probe Location, and the between-participant factor, Context. As shown below, this analysis revealed that there were systematic differences in endpoint hand angle between the three probe locations independent of the training environment, an effect that is most likely due to biomechanical biases. To compensate for these biases, we subtracted out the Base3 endpoint hand angles from the comparable values in the generalization blocks (see below).

To quantify learning of the visual perturbations, the endpoint hand angles of the last five trials during the Training block were averaged. We performed a two-step analysis with these data. First, we compared these values to 30° to determine if participants were fully adapted to the perturbation. Second, we conducted a two-sample *t*-test to determine if there were significant differences between the groups. In addition, we fit each participants' time series of hand angles in the training block with an exponential function using the Levenberg-Marquardt method for nonlinear least squares. To determine if there were differences in learning between the groups in Experiments 1 and 2, these values were also submitted to a two-sample *t*-test. The alpha value was set to 0.05 when only one test was performed and set to 0.025 when we performed a two-step analysis.

To assess generalization, we focused on the three probe locations in the Test block. The endpoint hand angle at each probe was calculated for each participant and the Base3 endpoint hand angles on trials without feedback were subtracted from these values. We then performed a two-step analysis. The first analysis was to determine if there was significant generalization at the probe locations within each group (differences greater than zero). The second analysis compared the endpoint hand angle data for the probe locations between the two groups. We also analyzed the Training and Test block data in a between-experiment supplemental analysis to directly compare performance in Experiments 1 and 2, using a two-way ANOVA with the factors Context and Perturbation.

The data from the No Feedback block provides a picture of the full generalization function. While we present these data qualitatively, our statistical analysis was restricted to two subregions. One subregion was composed of target locations near the training location. This included the target locations at 45° and −45° with the circular context and the target locations at 35.2° and −35.2° with a rectilinear context. The second subregion was composed of the three probe locations (circular context: 135°, 180°, and −135°; rectilinear context: 144.7°, 180°, and −144.7°). For each subregion, we performed the two-step analysis described above, again subtracting out the endpoint hand angles from the Base3 block. We did not statistically evaluate performance for the other targets (circular context: 90° and −90°; rectilinear context: 54.7° and 125.3°) because these locations did not have corresponding target locations within the other context.

## Results

### Experiment 1

Prior to the introduction of the visual perturbation, participants generally reached straight toward the target locations, terminating their movements just past the targets. The two exceptions were the 54.7° and 125.3° target locations in the rectilinear context, where the participants tended to not pass entirely through the targets because this was near the extent of a comfortable reach distance for these locations. Endpoint hand angle during the Base3 block did not differ between the groups [*t*_(14)_ = 1.65, *p* = 0.12]. The added movement distance required to reach the targets in the rectilinear context did not lead to a significant increase in movement time [*t*_(14)_ = 1.11, *p* = 0.28]. In fact, the trend was in the opposite direction, with average movement times of 280 ± 79 ms and 228 ± 28 ms for the circular and rectilinear contexts, respectively. The mean reaction time was 368 ± 31 ms in the circular context and 452 ± 59 ms in the rectilinear context, values that were significantly different [*t*_(14)_ = 2.47, *p* = 0.03].

As described in the Methods, we performed a restricted analysis on the three probe locations for the Base3 data. A mixed-model, repeated measures ANOVA revealed a significant effect of Probe Location [*F*_(2, 28)_ = 2.47, *p* = 0.03]. Reach trajectories toward the −135° and −144.7° target locations terminated slightly CCW relative to the target, while reaches toward the 135°, 144.7°, and 180° locations terminated slightly CW relative to the target. The effect of Context was not significant [*F*_(1, 7)_ = 0.02, *p* = 0.89], nor was the interaction of these two factors [*F*_(1, 7)_ = 1.33, *p* = 0.27]. Given that the probe location differences were independent of context, we assume that the effect reflects biomechanical biases associated with different limb configurations required for the different target locations. To minimize the effect of this bias in the subsequent analyses, we subtracted the Base3 endpoint hand angles to these three locations from the endpoint hand angles in the generalization blocks (see below).

**Figure 3:**
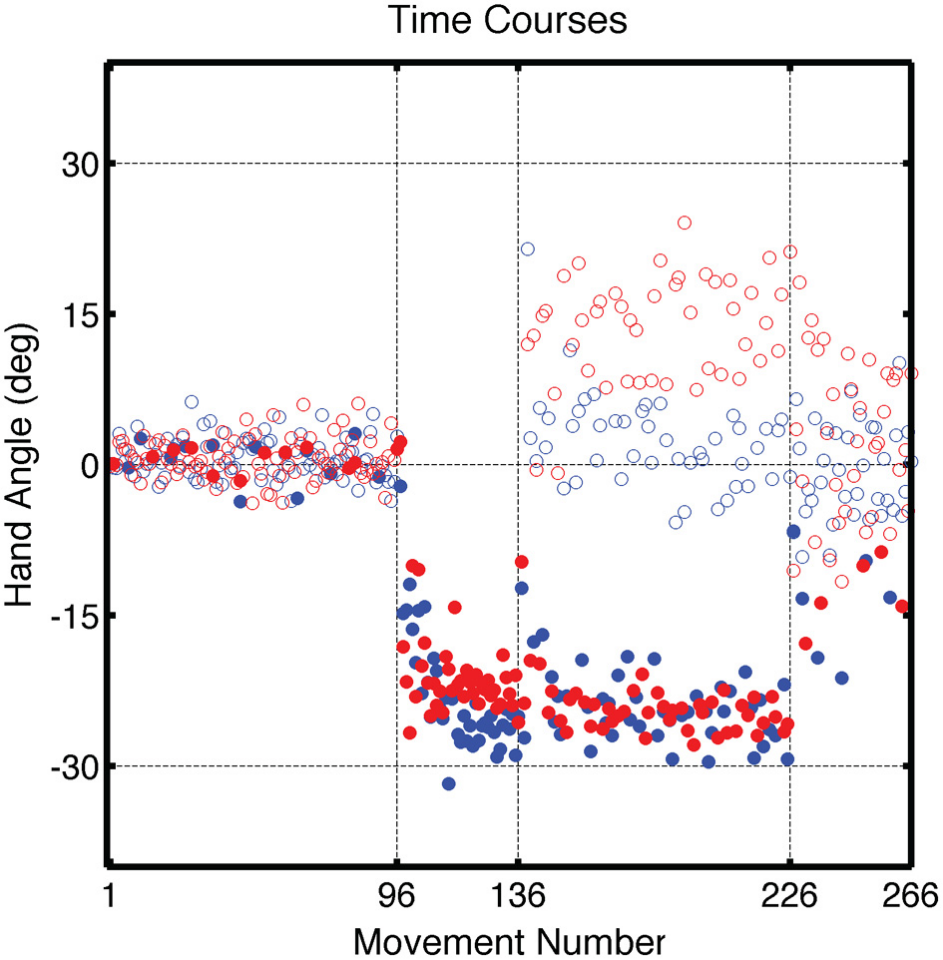
**Group averaged endpoint hand angle across trials in Experiment 1.** The visuomotor mapping was veridical for the first 96 trials (Base1, Base2, Base3). Dashed vertical lines mark when the visual perturbation was present during the Training block (movements 97–136) and during the Test block (movements 137–226). Filled circles represent movements to the training target location and open circles represent movements to other target locations (blue: CircleRotation group; red: LineTranslation group). Endpoint position for the LineTranslation group was converted from Cartesian to polar coordinates since the visual perturbation was identical in polar space for the two groups.

During the Training block, participants in both groups altered their movement trajectories to compensate for the perturbation (Figure 3). The average endpoint hand angle for the last five movements in the Training block was −25.9 ± 4.4° for the CircleRotation group and −22.9 ± 3.7° for the LineTranslation group. These values indicate that adaptation was not complete [CR: *t*_(7)_ = 2.66, *p* = 0.03; LT: *t*_(7)_ = 5.50, *p* < 0.001], with the average position of the feedback cursor falling below the target in both conditions. Nonetheless, this level of learning was similar between the groups [*t*_(14)_ = 1.47, *p* = 0.16]. To assess the overall learning functions, the time series of endpoint hand angles was fit with an exponential function. No differences were found between the CircleRotation and LineTranslation groups in the rate of learning [*t*_(14)_ = 1.10, *p* = 0.29], the final asymptotic level of learning [*t*_(14)_ = 1.99, *p* = 0.07], or the magnitude of learning [*t*_(14)_ = 1.02, *p* = 0.33].

During the Test block, reaches to the training target location were interspersed with reaches to the three probe locations. Visual feedback was presented on reaches to the training target and was withheld on reaches to the probe targets. For the training target location, participants continued to compensate for the visual perturbation. For the CircleRotation group, the average reach angle was −19.0 ± 3.5° over the first five movements of the Test block, which was significantly less than at the end of the Training block [*t*_(7)_ = 3.01, *p* = 0.02]. Participants in the LineTranslation group also showed a significant reduction in adaptation with an average reach angle of −19.5 ± 3.4° over the first five movements of the Test block compared to the end of the Training block [*t*_(7)_ = 3.28, *p* = 0.01]. We attribute the reduced adaptation to the fact that there was a short set break (approximately 30 s) between the end of the Training block and start of the Test block. Importantly, however, there was no significant difference between the two groups over these first five movements toward the training target [*t*_(14)_ = 1.15, *p* = 0.26] nor over the last five movements [*t*_(14)_ = 1.36, *p* = 0.19].

**Figure 4:**
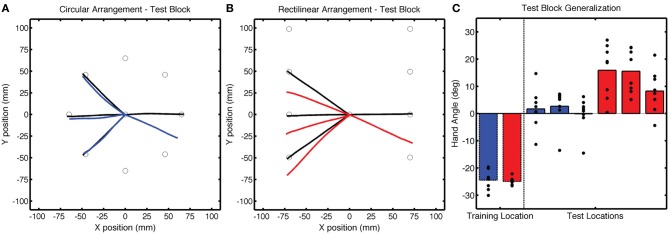
**Group averaged trajectories during the Test block in Experiment 1 for the (A) CircleRotation group (blue) and (B) LineTranslation group (red) compared with the averaged trajectories during the last baseline block (black). (C)** Mean endpoint hand angles for the training location and three probe target locations (135°, 180°, and −135°). Black circles represent the values for each participant.

Of greatest interest in terms of generalization is the participants' performance when reaching to the three probe locations. These data are presented in Figure 4 as colored lines (blue = CircleRotation; red = LineTranslation), alongside the trajectories to the same targets in the Base3 block (black). We subtracted the Base3 endpoint hand angle data from each value and performed a two-step analysis, first asking if the trajectories within each group deviated from a straight path to the targets (after correcting for the biases observed in Base3), and then comparing the two groups. For the CircleRotation group, generalization at the probe target locations was not significant [*t*_(7)_ = 0.65, *p* = 0.53]. In contrast, generalization was significant for the LineTranslation group [*t*_(7)_ = 4.89, *p* = 0.002]. When the data for the two groups were directly compared, generalization for the LineTranslation group was significantly greater than for the CircleRotation group [*t*_(14)_ = 3.38, *p* = 0.005].

**Figure 5:**
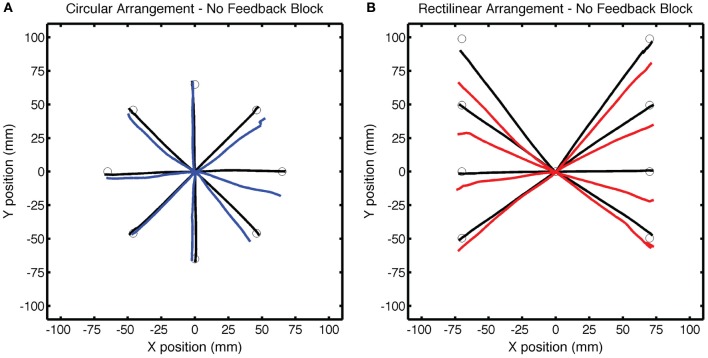
**Group averaged trajectories during the No Feedback block in Experiment 1 for the (A) CircleRotation group (blue) and (B) LineTranslation group (red) compared with the averaged trajectories during the last baseline block (black).** Movements to each target location were made without visual feedback.

The No Feedback block provided a picture of the full generalization function (Figure 5). The statistical analysis, however, was restricted to two subregions, one selected to be far from the training target location (the three probe locations), and one selected to be near the training target location (the two adjacent locations). Again, the Base endpoint hand angles were subtracted out to remove systematic biases at each target location. Generalization was significant at locations near the training location for both the CircleRotation group [*t*_(7)_ = 3.13, *p* = 0.02] and the LineTranslation group [*t*_(7)_ = 4.90, *p* = 0.001], and the degree of generalization was similar between the two groups at these near locations [*t*_(14)_ = 0.50, *p* = 0.62]. For the far locations, the pattern of generalization was similar to what had been observed in the Test block. Generalization was significant for the LineTranslation group [*t*_(7)_ = 3.05, *p* = 0.02], but not for the CircleRotation group [*t*_(7)_ = 0.94, *p* = 0.38]. When the two groups were directly compared, the difference was only marginally significant [*t*_(7)_ = 1.91, *p* = 0.08]. Note that, while we did not observe generalization at the probe locations for the CircleRotation group, the small shifts were actually in the opposite direction from what would be expected if participants had learned a rotation.

As can be seen in a comparison of Figures 4 and 5, the magnitude of generalization at the probe locations is weaker in the No Feedback block compared to the Test block. This result is expected given that adaptation decays over time in the absence of visual feedback (Hatada et al., 2006; Criscimagna-Hemminger and Shadmehr, 2008; Huang and Shadmehr, 2009).

### Experiment 2

The results of Experiment 1 suggest that there is broader generalization for a Cartesian translation compared to a polar rotation when each perturbation is presented in a context consistent with its respective perturbation. However, it is unclear if the broader generalization in the former condition is due to the type of perturbation (translation vs. rotation), the context (rectilinear vs. circular), or a combination of these factors. In Experiment 2, we examined these hypotheses by swapping the contexts for the two types of perturbations. One group (CircleTranslation) was presented with a translational perturbation when reaching to targets arranged in a circular context while a second group (LineRotation) was presented with a rotation when reaching to targets arranged in a rectilinear context (Figure 2).

During the Base3 block, there were no significant differences between the groups: participants showed similar target errors [*t*_(14)_ = 1.48, *p* = 0.16], reaction times [*t*_(14)_ = 0.12, *p* = 0.27], and movement times [*t*_(14)_ = 0.75, *p* = 0.46], indicating that the contexts did not affect reaching behavior in the absence of a visuomotor perturbation. When the heading analysis was restricted to the three probe locations, we again observed a significant effect of Probe Location [*F*_(2, 28)_ = 9.48, *p* = 0.001], but no effect of Group [*F*_(1, 7)_ = 5.65, *p* = 0.76] or interaction of these factors [*F*_(2, 14)_ = 0.43, *p* = 0.52]. Participants exhibited a similar bias pattern to that observed in Experiment 1.

**Figure 6:**
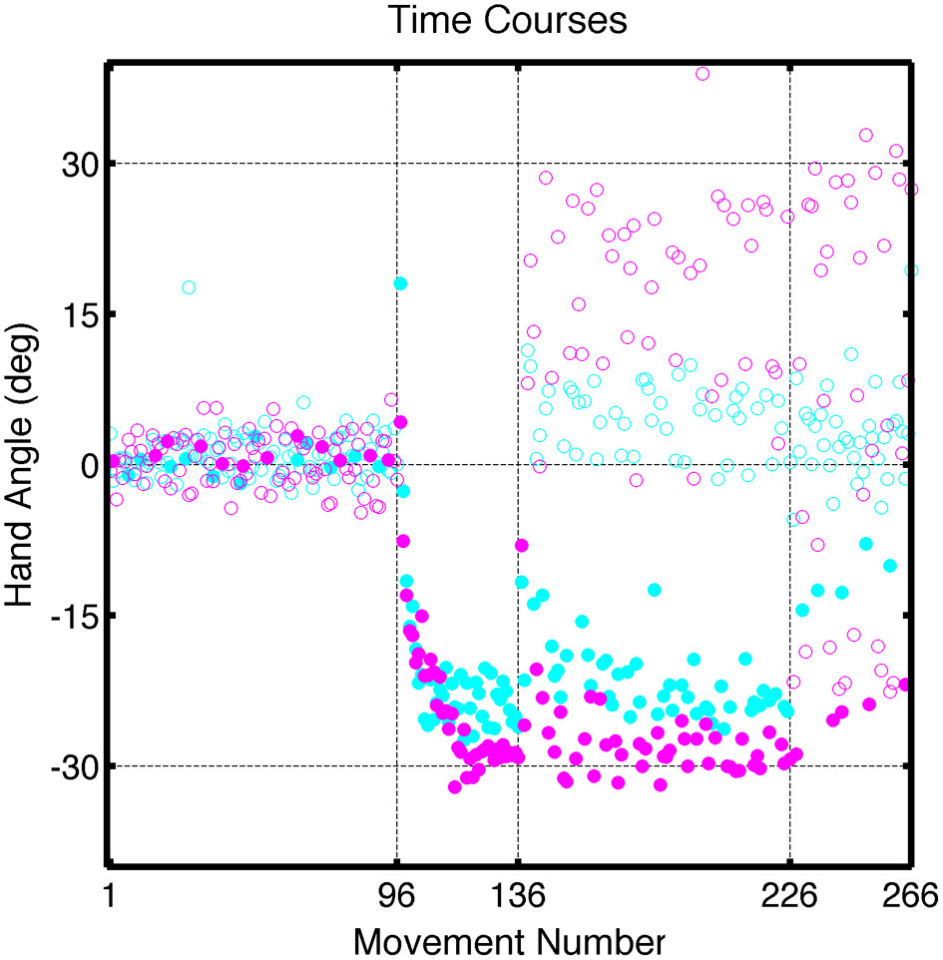
**Group averaged endpoint hand angle across trials in Experiment 2.** Block structure was the same as in Figure 2. Filled circles represent movements to the training target location and open circles represent movements to other target locations (cyan: CircleTranslation group; purple: LineRotation group).

Participants showed rapid learning of both visual perturbations (Figure 6). The average endpoint hand angles over the last five trials during the Training block were −24.7 ± 2.47° for the CircleTranslation group and −28.9 ± 3.00° for the LineRotation group, values that were significantly different [*t*_(14)_ = 3.02, *p* = 0.01]. Compared to the value corresponding to full adaptation (30°), the CircleTranslation group showed incomplete learning [*t*_(7)_ = 6.05, *p* < 0.001]. This comparison was not reliable for the LineRotation group [*t*_(7)_ = 1.07, *p* = 0.32], a null result consistent with complete learning. An exponential fit of the time series of endpoint hand angles also revealed a difference in the asymptotic level of learning between the groups [*t*_(14)_ = 2.30, *p* = 0.04], consistent with greater learning in the LineRotation group. However, the groups did not differ in terms of learning rate [*t*_(14)_ = 1.27, *p* = 0.23] or magnitude of learning [*t*_(14)_ = 1.00, *p* = 0.34].

**Figure 7:**
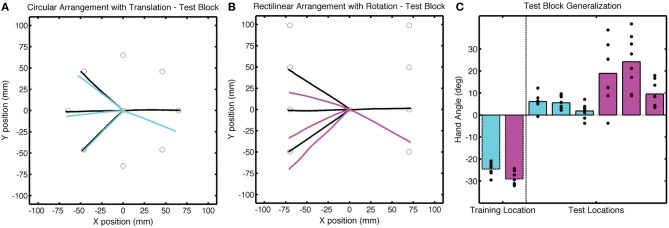
**Group averaged trajectories during the Test block in Experiment 2 for the (A) CircleTranslation group (cyan) and (B) LineRotation group (purple) compared with the averaged trajectories during the last baseline block (black). (C)** Mean endpoint hand angles for the training location and three probe target locations (135°, 180°, and −135°). Black circles represent the values for each participant.

Despite the subtle performance differences during the training block, we observed dramatic differences in generalization at the probe locations in the Test block (Figure 7). After correcting for the Base3 biases, significant generalization was observed for both the CircleTranslation group [*t*_(7)_ = 4.96, *p* = 0.002] and the LineRotation group [*t*_(14)_ = 6.88, *p* < 0.001]. However, the magnitude of generalization was considerably larger in the LineRotation group [*t*_(14)_ = 4.82, *p* < 0.001]. Note that this increase in generalization was observed despite the fact that this group had shown less adaptation during the training phase.

**Figure 8:**
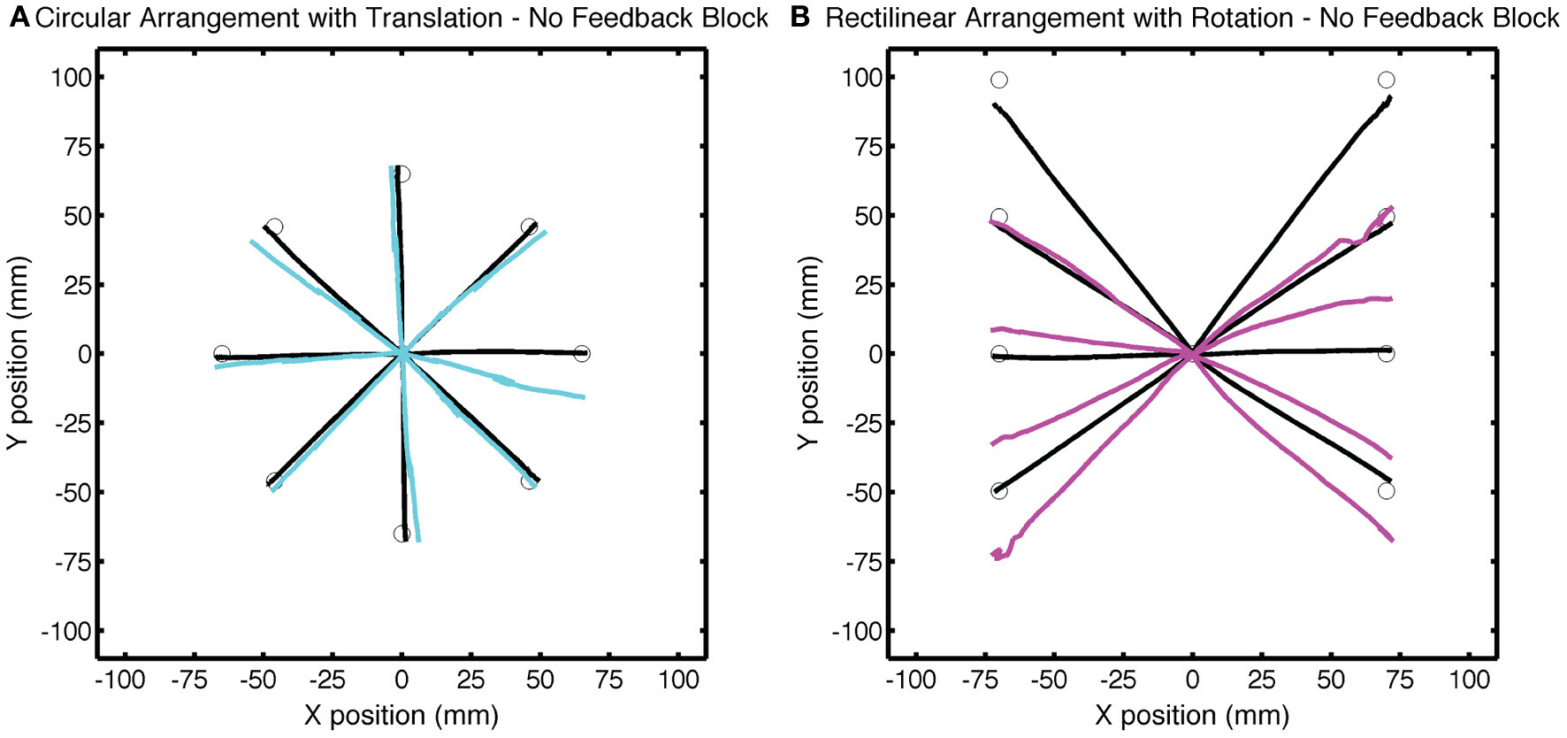
**Group averaged trajectories during the No Feedback block in Experiment 2 for the (A) CircleTranslation group (cyan) and (B) LineRotation group (purple) compared with the averaged trajectories during the last baseline block (black).** Movements to each target location were made without visual feedback.

Differences in the amount of generalization between the two groups was also evident in the No Feedback block, with larger changes in hand angle for the LineRotation group at both near and far locations (Figure 8). For the CircleTranslation group, small but significant generalization was observed for both the near target locations [*t*_(7)_ = 2.71, *p* = 0.03] and probe target locations [*t*_(7)_ = 3.88, *p* = 0.006]. Generalization was also reliable at both subregions for the LineRotation group [near: *t*_(7)_ = 9.01, *p* < 0.001; far: *t*_(14)_ = 7.78, *p* < 0.001]. When the two groups were compared, the LineRotation group exhibited larger generalization for the near [*t*_(14)_ = 5.65, *p* < 0.001] and far [*t*_(14)_ = 5.85, *p* < 0.001] subregions.

It is important to note that the direction of generalization was similar for both groups, with the trajectories shifted in the downward direction. This would be expected if participants were learning an internal model of a translation shift since compensation for the upward shift requires a downward displacement of the trajectory. However, it is opposite of what would be expected if participants were learning an internal model of a rotation. Generalization of a rotation to the probe locations would require an upward displacement of the trajectories to these targets.

To directly compare the data from Test blocks in the two experiments, we employed a two-way ANOVA with the factors Context (circular vs. rectilinear) and Perturbation (rotation vs. translation). There were no significant effects in the degree of adaptation at the training target location during the training block, although both main effects and the interaction approach significance [Context: *F*_(1, 14)_ = 2.69, *p* = 0.11; Perturbation: *F*_(1, 14)_ = 1.17, *p* = 0.29; interaction: *F*_(1, 7)_ = 1.44, *p* = 0.24]. In terms of generalization, only the effect of Context was significant [*F*_(1, 14)_ = 33.6, *p* < 0.001]. The type of perturbation was not significant [*F*_(1, 14)_ = 0.18, *p* = 068], nor was the interaction of these factors [*F*_(1, 14)_ = 2.34, *p* = 0.14]. When averaged across the three probe locations and between experiments, the mean shifts in hand angle were 15.4 ± 7.5° and 2.66 ± 4.7° for the rectilinear and circular contexts, respectively. Thus, when the two experiments are considered together, the results clearly demonstrate that generalization, at least to targets far from the training location, is constrained more by the context rather than the error information.

### Experiment 3

In Experiments 1 and 2, the visual perturbations were introduced abruptly and participants were likely cognizant at the beginning of the training block that their performance was no longer accurate. It is possible that learning with rectilinear context induced the adoption of a generic strategy rather than the adaptation of an internal model (Taylor and Ivry, 2012). For example, the participants may have noticed that the reaches were terminating above the target location and decided to aim to a location below the target. Generalization would appear broad if this strategy was applied to all of the targets. By this hypothesis, the results of Experiments 1 and 2 would indicate that the rectilinear arrangement leads participants to adopt a strategy, whereas the circular arrangement does not. Alternatively, it may be that the rectilinear arrangement produces greater sensorimotor adaptation than the circular arrangement.

To assess these two hypotheses, we employed a procedure that has been used to prevent strategy use in previous studies of visuomotor adaptation. Instead of introducing the perturbation abruptly, a small, incremental perturbation was introduced over the course of an extended, 160-trial Training block. Under such conditions, participants exhibit minimal, if any, awareness of the perturbation (Malfait and Ostry, 2004; Saijo and Gomi, 2010; Taylor et al., 2011). Given that our focus here is to understand why the rectilinear context produces broad generalization, we only tested one group of participants (*n* = 8), using the LineTranslation condition in the rectilinear context. The translational shift was introduced in small increments, increased linearly from 0 cm to 3.5 cm over the course a 160 trial training block (a shift of 0.023 cm or 0.188° per trial for the first 152 trials, then held constant over the last 8 trials). The remaining structure of the baseline blocks and generalization blocks was the same as in Experiments 1 and 2, resulting in a total of 386 movements.

**Figure 9:**
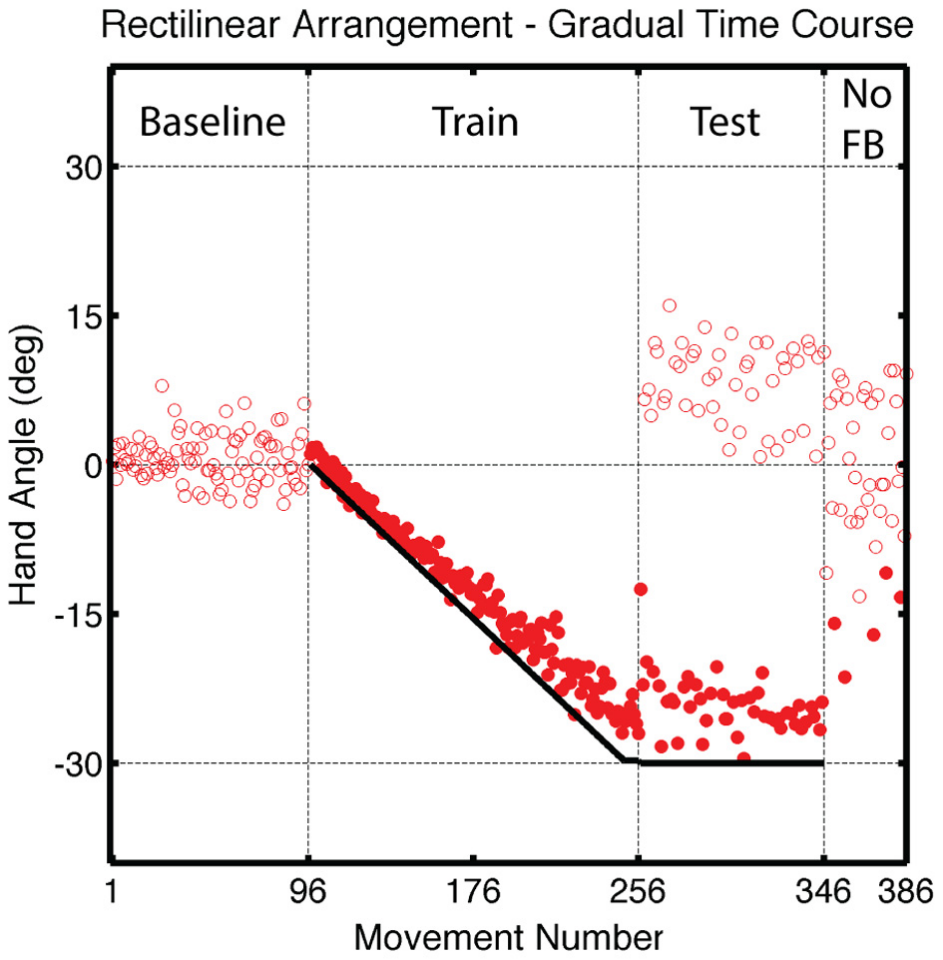
**Group averaged endpoint hand angle across trials in Experiment 3.** A vertical shift (black solid line) was introduced in an incremental manner during the Training Block, reaching a final value of 4 cm (movements 97–256). Feedback was only provided for reaches to the training target location during the Test block (movements 256–226). Visual feedback was never provided in the final, No Feedback block. Filled circles represent movements to the training target location and open circles represent movements to other target locations.

Participants learned to offset the gradual perturbation during the Training block (Figure 9). Over the last five trials, the average endpoint hand angle was −25.1 ± 1.96°, which fell short of complete learning of the 30° perturbation [*t*_(14)_ = 7.08, *p* < 0.001]. Since the perturbation was introduced linearly during the Training block, we used a linear function to fit the time course data. The observed slope of 0.17 ± 0.02° per trial was slightly less than the 0.188° slope of the perturbation function [*t*_(7)_ = 52, *p* < 0.001].

**Figure 10:**
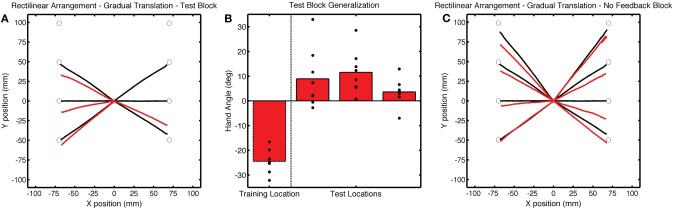
**(A)** Group averaged trajectories during the Test block (red) in Experiment 3. **(B)** Mean endpoint hand angles for the training location and the three probe target locations (135°, 180°, and −135°). Black circles represent the values for each participant. **(C)** Average trajectories during the No Feedback block. Black lines in **(A)** and **(C)** correspond to average trajectories during the last baseline block.

While participants continued to compensate for the translational shift when reaching to the training target location during the Test block, there was a initial decrease in hand angle (Figure 9), likely due to decay during the transition between the Training and Test blocks. Over the first five movements to the training target, the average hand angle was −19.5 ± 1.79°, which was less than that observed in the last five trials of the Training block [*t*_(7)_ = 5.92, *p* < 0.001]. Generalization at the probe locations was observed in the Test block [*t*_(7)_ = 2.96, *p* = 0.02; Figures 10A,B] and was also evident across the workspace in the No Feedback block [near targets: *t*_(14)_ = 5.49, *p* < 0.001; far, probe targets: *t*_(14)_ = 3.18, *p* = 0.02; Figure 10C]. Indeed, the magnitude of generalization at the probe locations was similar to that observed for the LineTranslation groups in Experiment 1 in a between-experiment comparison [*t*_(14)_ = 1.35, *p* = 0.20].

It is possible that the increased error at the beginning of the Test block may have led to some awareness of the perturbation. However, there are a few reasons why we do not think that this led to the observed pattern of generalization. First, generalization at the probe locations was apparent at the start of the Test block, measured over the average of the first five reaches [*t*_(7)_ = 5.92, *p* < 0.001]. Second, while post-experiment questionnaires revealed that most participants had a general sense that there was some sort of experimental manipulation of the feedback, they were not able to able to articulate the manipulation and reported reaching straight toward the probe target location. Third, if the generalization was due to the generic application of a strategy, we would expect it to be of similar magnitude as that observed at the training location; however, the results show that is was considerably attenuated. In sum, the results of Experiment 3 indicate that broad generalization for the rectilinear context was not dependent on conditions that might induce the use of a strategy.

## Discussion

### Summary

The set of experiments presented here highlight an important constraint on generalization following sensorimotor adaptation. Experiment 1 replicated previous work, showing that generalization was much broader when a visuomotor perturbation involved a translational shift compared to a rotation. Previous accounts of this difference have attributed it to how the type of perturbation, and its resultant error, is used to update an internal model (Thoroughman and Taylor, 2005; Hinder et al., 2008; Shabbott and Sainburg, 2010; Taylor et al., 2013). However, the results of Experiment 2 provide compelling evidence that the training context is the primary factor underlying this difference. Broad generalization was observed with a rotation when the targets were in a rectilinear arrangement, and became much smaller for a translation when the targets were arranged in a circular arrangement. The broad generalization pattern for the line context also held when participants were largely unaware of the visual perturbation (Experiment 3). Taken together, these results show that the pattern and breadth of generalization is strongly constrained by the training environment.

### The role of error signals in models of generalization

In examining generalization, researchers have focused on the breadth of the generalization function and the reference frame in which generalization is expressed. Across a number of studies, a picture has emerged in which the pattern of generalization varies for different visual perturbations. Generalization of rotations has been shown to be quite narrow, with the modest generalization at distant locations showing trajectory deviations that are consistent with the rotation. For example, with a clockwise rotation, the small amount of generalization for probe locations 180° from the training location are also in the clockwise direction (Pine et al., 1996; Krakauer et al., 2000). In contrast, generalization following gain changes (Krakauer et al., 2000; Pearson et al., 2010) and translational perturbations is quite broad, with the latter evident following prism adaptation (Bedford, 1993) or cursor shifts in one Cartesian dimension (Ghahramani et al., 1996). Thus, for translational perturbations, the trajectory deviations are consistent with what would be expected if participants had learned to compensate for a translation (Ghahramani et al., 1996). However, recent studies have suggested that generalization may entail multiple components, and that these may be expressed in multiple reference frames (Brayanov et al., 2012; Taylor et al., 2013).

Differences in generalization have been attributed to the error signal caused by the perturbation (Krakauer et al., 2000; Taylor et al., 2013). In a previous study involving a rotation, we found that systematically increasing the quality of visual error information led to different patterns of generalization (Taylor et al., 2013). When online feedback was provided throughout the entire movement, generalization was manifest as trajectory deviations that would suggest the participants had learned a rotational perturbation. In contrast, when feedback was limited to knowledge of results (endpoint feedback), the trajectory deviations were in the opposite direction, consistent with what would be expected if the participants had inferred a translational perturbation. Intermediate levels of feedback led to reference frame effects that fell between that observed with full online and endpoint only feedback.

Computational models of adaptation have employed radial basis function networks to explain how error signals are used to update a sensorimotor mapping and to explore the constraints on generalization (Thoroughman and Shadmehr, 2000; Thoroughman and Taylor, 2005; Tanaka et al., 2009; Pearson et al., 2010). The basis function network provides a representation of movement direction, through the weighted sum of individual units that are tuned to a particular movement direction. As such, the network activity results in a population vector, with each unit voting for a preferred direction of movement (Georgopoulos et al., 1986). Gradient descent is used to update the weight of each unit, with the change a function of the degree of an unit's activity level and the size of the visual error signal. Narrow generalization arises in this model because the tuning function serves as a weight on the error signal; that is, the effect of adaptation is greatest for units that are active at the time that the error is experienced. A critical feature of our model for generalization is that the different feedback conditions afforded different opportunities for error-driven learning. Endpoint feedback, with its discrete feedback, provided only a single opportunity for updating the internal model. In contrast, we proposed that online feedback provided additional opportunities for updating. Thus, we modeled conditions with online feedback corrections or movements that returned to the target with a second update during each trial. Here the rotational errors were experienced when units were active with a directional tuning quite different from those active when initially reaching to the training target (e.g., if during a return movement, the active units would be in the opposite direction). In this manner, adaptation could occur across a broad set of the basis functions, providing a mechanistic account of generalization.

A key insight from this work is that differences in the pattern of generalization between the feedback conditions were not necessarily inherent to differences in tuning functions, but rather an incidental by-product of the state of the network at the time of error updates. For example, a counterclockwise rotation during the outbound portion of a movement (see Figure 1) would adjust units tuned toward 0° in the clockwise direction. The same rotation during the return movement, would also produce a shift in the clockwise direction, but here the effect is on units tuned toward 180°. Thus, when generalization is tested for movements around 180°, the trajectories would exhibit a clockwise shift, suggesting that the participants had learned a rotation. However, the model suggests that this is not generalization *per se*, but rather the incidental effect of local adaptation for movements in this direction. That is, generalization with online feedback is the composite effect of multiple local adaptation effects.

An alternative perspective on the difference between translational and rotational generalization focuses on the reference frame within which learning occurs. A translation can be viewed as a perturbation defined in an extrinsic reference frame; for example, the displacement of a soccer kick from any point on the field will be affected in a similar manner by a strong wind. The reference frame for a rotation is more ambiguous. It could be in extrinsic space, defined by polar coordinates. Or it could be defined intrinsically as has been shown in force field adaptation where learning generalizes in joint space (Shadmehr and Mussa-Ivaldi, 1994).

It is important to note that in the current study, as well as several other studies of visuomotor adaptation, the reference frame of learning cannot actually be inferred from the pattern of generalization. Generalization always appeared to be translational, consistent with the idea that it operates in an extrinsic reference frame. However, it remains unclear if this pattern reflects the reference frame of learning, or if it reflects an inference about the nature of the perturbation. With endpoint feedback, the motor system may be unable to infer the precise nature of the perturbation and a translation may be the default inference, even with a rotational perturbation. With online feedback, a rotational perturbation would result in curved trajectories. This may bolster an inference that the perturbation is, in fact, rotational. Insight into the reference frame of learning can be gained by having participants reach to the same set of target locations, but with an altered limb configuration during generalization trials. Using this approach, Brayanov et al. (2012) found that generalization of a rotation entailed a mixture of multiple reference frames. It remains an open question if a rotation induces adaptation in multiple reference frames, or if performance reflects a mixture of multiple inferences about the nature of the perturbation.

### The influence of context

Independent of the reference frame debate, the present results pose a problem for current models of generalization. It does not seem likely that context would affect low-level representations of movement, such as the tuning function of the units in the basis function model. The current results indicate that a full model of generalization must go beyond consideration of tuning functions and error signals, incorporating the influence of context in how participants make inferences about the nature of the error signal. There are two related issues to keep in mind here. First, and most compelling, generalization was much more substantial at the probe locations with the rectilinear context compared to the circular context. Second, for a given context, there was little difference between the two types of perturbations: Generalization was broad and substantial for the rotational and translational perturbations with a rectilinear context, and minimal for both types of perturbations for the circular context.

There are various ways in which to consider this contextual effect. One idea is that both the error and context define the reference frame for learning. For example, the rectilinear context may promote a conceptualization that is extrinsic or world based, whereas the circular context may promote a conceptualization that is intrinsic or body based. If the error signal is always in extrinsic coordinates, then the context and error both converge on a common, extrinsic reference frame. In contrast, the context and error would be in opposition for a circular context. By this view, the minimal generalization seen at distant locations with the circular context is due to the canceling effects of the two factors, whereas the broad generalization at these locations with the rectilinear context is due to their complementary effects.

A second idea relates back to the idea that generalization may be captured by a mixture of experts model (Ghahramani and Wolpert, 1997; Krakauer et al., 2000; Pearson et al., 2010), one form of which is reflected in models in which generalization involves a combination of local and global components. In these models, the perturbation may be learned by modular decomposition by expert modules at a very local level (as with direction tuned units), and then combined with a weighting, or gating function to account for generalization that is manifest at the global level (Ghahramani and Wolpert, 1997; Pearson et al., 2010). In current versions of this model, adaptation of the local modules is based on the size of the error signal, regardless of context. Indeed, this weighting idea has previously been considered in terms of the how translational and rotation error signals might produce different patterns of generalization (Ghahramani et al., 1996; Ghahramani and Wolpert, 1997; Krakauer et al., 2000; Pearson et al., 2010). However, context may change how these local units are combined at the global level. By this hypothesis, the effect of context could be viewed, not in terms of how it influences the reference frame of generalization, but rather in terms of how it constrains the weighting function. One potential problem for a simple weighting function model is that learning at the training location and generalization to the near probes was similar for the two contexts. As such, it cannot be that the rectilinear context simply produced an overall increase in the weighting function. Rather, a two-process model would be required, one in which local adaptation is based on the error signal independent of context, and a second in which context constrains how that information is broadcast globally.

Why might the motor system give less weight to a circular context (and rotational perturbation)? One hypothesis is that the weighting function is modulated by perturbation uncertainty. A rotational perturbation is inherently nonlinear and more complex than a translational perturbation. Because of this complexity, the participant is more uncertain about the perturbation. Increased uncertainty may attenuate the weighting function, resulting in weaker generalization at distant locations. While the uncertainty idea could be considered with respect to the error signal, the current results make clear that the weighting hypothesis must be modified to consider context as a key constraint. Specifically, the arrangement of the targets may provide clues to the motor system as to the nature of the perturbation. A linear arrangement of the environment could bias the system to infer a linear solution to offset the perturbation. A circular arrangement of the environment could bias the system to infer a more complex, non-linear solution. As with the error-based models, this more complex (or ambiguous) environment results in an attenuated weighting function due to uncertainty.

We recognize that we are only offering speculative ideas about the mechanisms through which context influences generalization. We do believe the ideas outlined here provide a framework for considering constraints on motor learning, with the key insight that the context must be part of the equation. Future experiments could better manipulate how the combination of information in the error signal and the training environment guide learning and generalization. Ultimately, the motor system is faced with an inductive inference problem, especially when sensory information is limited, to make predictions about the underlying state of the world. The error signal and the context within which that information is presented are exploited to best resolve an ambiguous inference problem.

### Conflict of interest statement

The authors declare that the research was conducted in the absence of any commercial or financial relationships that could be construed as a potential conflict of interest.
